# Evaluation of Talimogene Laherparepvec for the Treatment of Advanced Nonmelanoma Skin Cancers

**DOI:** 10.1245/s10434-026-19182-3

**Published:** 2026-02-22

**Authors:** J. Slatton, V. Jiminez, J. Beam, N. Rademacher, K. Montgomery, S. Bateni, K. Broman

**Affiliations:** 1 Heersink School of Medicine, University of Alabama at Birmingham, Birmingham, AL USA; 2 Department of Surgery, University of Alabama at Birmingham, Birmingham, AL USA; 3 Institute for Cancer Outcomes and Survivorship, University of Alabama at Birmingham, Birmingham, AL USA; 4 Department of Surgical Oncology, University of Texas MD Anderson Cancer Center, Houston, TX 77030 USA

**Keywords:** Oncolytic virus therapy, Talimogene laherparepvec, Non-melanoma skin cancer, Outcomes research, Retrospective cohort

## Abstract

**Introduction:**

Talimogene laherparepvec (T-VEC) is an intralesional, injectable oncolytic herpes virus that is FDA-approved to treat Stage III-IV melanoma. The utility of T-VEC for nonmelanoma skin cancers (NMSC), including Merkel cell carcinoma (MCC) and squamous cell carcinoma (SCC), as well as its use with concurrent immune therapy (IO), is poorly defined. The purpose of this study is to evaluate outcomes of T-VEC for MCC and SCC with and without concurrent immune therapy.

**Methods:**

This retrospective study included patients at a single institution with MCC or SCC and treated with T-VEC between May 2016 and May 2023. Abstracted cohort data were reported using descriptive statistics.

**Results:**

Ten MCC and three SCC patients were eligible. Four MCC patients and all three SCC patients received concurrent pembrolizumab. Overall, six patients achieved complete response (CR), one had partial response (PR), and six had progressive disease (PD). Of the six patients who achieved CR, two recurred at 8 and 56 months. Those without recurrence had a durable response at a median follow-up of 25 months.

**Conclusions:**

Our initial experience with a small, single-institution cohort demonstrated tumor response to intralesional T-VEC with or without immunotherapy in some patients with NMSC. Nearly 50% of participants had a complete response; two-thirds of responders remained relapse-free at the time of follow-up. Some observed responses are not attributable to T-VEC alone due to high usage rates of IO. Future work should expand to larger cohorts and focus on its use with and without immune therapy.

**Supplementary Information:**

The online version contains supplementary material available at 10.1245/s10434-026-19182-3.

Nonmelanoma skin cancer (NMSC), including squamous cell, basal cell, and Merkel cell carcinoma, is an increasingly common problem in the United States. From 2005 to 2019, the incidence of squamous cell carcinoma (SCC) and basal cell carcinoma (BCC) increased from 402 to 787 per 100,000 persons.^1^ From 2000 to 2013, Merkel cell carcinoma (MCC) also saw an increase in incidence from 0.5 to 0.7 per 100,000 persons.^2^ While most cases of NMSC present as localized disease amenable to surgical resection, some patients have advanced disease that cannot be cured by surgery.^1^ Treatment options are limited for these patients, who are often older, immune suppressed, and have additional comorbid conditions.

Intralesional therapies using oncolytic viruses represent one strategy for managing surgically unresectable cutaneous malignancies. Talimogene laherparepvec (T-VEC), a herpes simplex virus 1 (HSV-1)-based oncovirus, has demonstrated efficacy in treatment of Stage III and IV melanoma and received FDA approval for this indication in 2015.^3^ T-VEC is a modified version of the JS17 strain of HSV-1 in which neurovirulence genes have been replaced by the human granulocyte-macrophage colony-stimulating factor (hGM-CSF) gene, which can instigate an antitumor T-cell response.^3^ Additional modifications facilitate antigen-presentation and prolonged host response.^3^ These modifications help initiate and maintain an immune response that is more robust and specific to tumor cells, while minimizing adverse effects in nontumor tissues.^3^

There is reported use of T-VEC for NMSC, although results are mostly limited to single case reports and case series, and treatment details and outcomes are limited.^4–6^ Furthermore, prior work has largely examined the use of T-VEC in isolation or with conventional chemotherapy and radiation therapy, whereas concurrent treatment with systemic immune therapy has not been well-described. Given limited contemporary data on the use of T-VEC for NMSC, this study evaluated its use at an NCI-designated comprehensive cancer center whose Southeastern US referral base spans five states.

## Methods

This retrospective study cohort included adult patients who underwent intralesional therapy with T-VEC for advanced or refractory MCC or SCC at the University of Alabama at Birmingham (UAB), an NCI-designated Comprehensive Cancer Center in the Southeastern United States, from May 2016 to May 2023. Patients were identified from the institution’s Enterprise Data Warehouse (EDW) using diagnosis codes for NMSC (SCC = C44.0x; MCC = C4A.x), treatment codes (CPT = 96405, 96406), and physician orders for Talimogene laherparepvec. Participants received at least one dose of T-VEC for NMSC at the study institution.

T-VEC was administered per the standard treatment protocol for melanoma, consisting of up to 4 mL per dose.^7^ Dose was determined based on the total size of injected lesions, and more than one lesion could be injected at a time. After the initial injection, the second dose was administered 3 weeks after the initial dose, then every 2 weeks thereafter. Treatment continued until evidence of complete clinical response or definitive clinical progression. Complete response was defined as gross resolution of all clinically detected lesions, augmented by pathologic confirmation of absence of viable tumor in cases where posttreatment biopsy was performed. Partial response was defined as reduction in number and/or size of clinically detected lesions, and/or pathologic finding of both viable and nonviable tumor in any obtained specimens. Progressive disease was defined as increase in number and/or size of clinically detected lesions or newly identified distant site(s) of disease. Responses were evaluated by visual inspection and interval imaging (where applicable, if lesions visible by imaging) as reduction in size, number, and/or FDG avidity (where applicable) based on clinical measurements performed at 2-week intervals.

All data were generated during routine care and were abstracted secondarily for research purposes. Patient data were stored in REDCap, a secure, HIPAA-compliant database management software. Abstracted data included demographics, comorbid conditions (other cancers, immune suppression), prior treatment history, T-VEC administration details, including adverse events and response, and long-term outcomes, including disease and vital status. These data were ascertained retrospectively among eligible patients, none of whom were treated in the context of a clinical trial or protocol, and in all cases T-VEC was used after demonstrated refractoriness to standard therapies due to lack of therapeutic alternatives and considering the favorable risk profile in available trials in melanoma. Statistical analysis was performed in Stata 15.1. Descriptive statistics were used to describe T-VEC treatment duration, overall response rate (ORR), defined as number of participants with partial (PR) or complete response (CR) divided by number of participants treated, and disease status at follow-up (alive without disease, alive with disease, deceased from disease, deceased from other causes). Categorical variables were described using counts and proportions. Continuous variables were nonparametric and were described using medians with interquartile ranges.

The study was approved by the University of Alabama at Birmingham institutional review board (#3000006558).

## Results

The study cohort included 13 patients (10 patients had a diagnosis of MCC, 3 patients had a diagnosis of SCC) (Table 1). Due to the variable nature of treatment received prior to and after initiating T-VEC in our cohort, a summary of each participant’s clinical course has been provided in the supplemental table. All 13 participants had recurrent disease that was limited to in-transit sites and was refractory to alternative treatments, including surgery, radiation, chemotherapy, and, in some cases, immunotherapy (IO). Participants either received T-VEC alone (*N* = 6) or in conjunction with systemic IO with pembrolizumab (*N* = 7). Two of these seven patients, both with SCC, received pembrolizumab for 2.5 months and 6.5 months prior to initiating TVEC for 3 doses and 10 doses, respectively. One patient had T-VEC added due to progressive disease, whereas the other had it added to address a small amount of residual disease. The patient with progressive disease continued to progress on T-VEC, after which it was discontinued, whereas the patient with partial response to pembrolizumab achieved clinical complete response with addition of T-VEC. Among the five who started T-VEC concurrent with pembrolizumab, one with SCC and three with MCC had progressive disease. The remaining individual with MCC who started T-VEC concurrently with pembrolizumab achieved complete response. The median treatment duration was 4 doses, with a range of 1 to 15 doses.

**Table 1:** Cohort demographics

Variable	Cohort (*n*, %) (*N* = 13)
Age (years)	82 (74–87)
*Sex*	
Male	11 (84.6)
Female	2 (15.4)
*Race*	
White	12 (92.3)
Black/African American	1 (7.7)
Asian/Pacific Islander	0 (0)
Native American	0 (0)
Other/Unknown	0 (0)
*Ethnicity*	
Hispanic/Latino	0 (0)
Not Hispanic/Latino	13 (100)
*Insurance payor*	
Medicare	12 (92.3)
Medicaid	0 (0)
Private Insurance	1 (7.7)
Other insurance	0 (0)
Uninsured	0 (0)
*Primary tumor*	
MCC	10 (76.9)
SCC	3 (23.1)
Prior therapy*	
Surgery	12 (92.3)
Radiation	3 (23.1)
Chemotherapy	1 (7.7)
Immune therapy	7 (53.8)
*Received immune suppression^*	
Yes	2 (15.4)
No	11 (84.6)

Variables are reported as median (interquartile range) for continuous measures and number (percentage of cohort) for categorical measures

*N > 13 for “Prior Therapy” due to some patients receiving more than one treatment modality prior to T-VEC. Number in parentheses represent what portion of the cohort (out of 13) received said therapy

^Both patients with history of immune suppression had diagnoses of Crohn’s disease; one patient received adalimumab and azathioprine and the other received infliximab initially and transitioned to vedolizumab

**Fig. 1:**
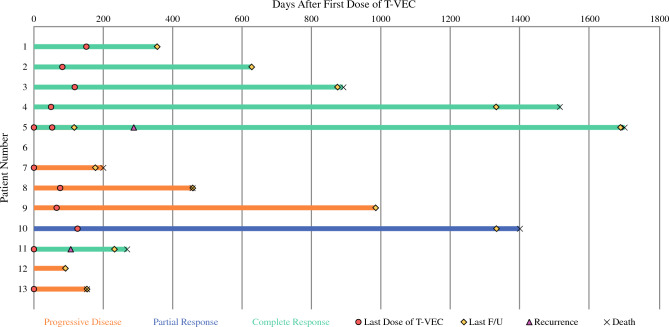
Swimmer plot showing the clinical course of each patient, including length of treatment, time spent in remission (if CR achieved), and dates of last follow-up and death when available

The ORR was 53.8% (7/13) (Table 2). Of these individuals, six had a clinical complete response (46.1%) and one had a partial response (7.7%). Six patients had progressive disease (46.1%). By tumor type, 60% of patients with MCC had a complete or partial response (1 with IO, 5 without IO). Among SCC cases, 33% had a complete or partial response (1 with IO, 0 without IO). Of the six individuals who achieved CR, four had continued response at a median follow-up of 25 months. Two of the six individuals with CR to T-VEC recurred at 8 and 56 months after cessation of T-VEC treatment (Fig. 1). No patients had Grade 3 or higher adverse events or discontinued T-VEC due to inability to tolerate treatment. The incidence of adverse events in the cohort are reported in Table 3. Available records show that eight patients (61.5%) are deceased.

**Table 2:** Summary of patient response rates stratified by type of disease and presence or absence of concurrent immune therapy (± IO)

	MCC		SCC		Total
	+ IO	− IO	+ IO	− IO	
Complete response (CR)	1	4	1	0	6
Partial response (PR)	0	1	0	0	1
Stable disease (SD)	0	0	0	0	0
Progressive disease (PD)	3	1	2	0	6
Total	4	6	3	0	13

**Table 3:** Clinically documented adverse events by treating providers

Adverse event	Number (%)
Flu-like symptoms, including fever, chills, and fatigue	5 (38.5)
Gastrointestinal symptoms, including nausea, vomiting, diarrhea	3 (23.1)
Confusion/altered mental status	1 (7.7)
Mild injection site reactions (pain, swelling, redness)	1 (7.7)

## Discussion

In this institutional cohort of patients who underwent intralesional T-VEC therapy with or without systemic immunotherapy for the treatment of recurrent, unresectable local or in-transit NMSC, more than 50% of patients had a partial or complete response. Among responders, most were durable at a median follow-up greater than 2 years.

Patients with recurrent and/or in-transit MCC and SCC represent a clinical challenge for which alternative therapies are needed. Many of these patients have previously undergone surgery and/or radiation and are not candidates for additional operations or re-irradiation. A 2014 study by Satpute et al. investigated 12 patients who received platinum-based chemotherapy for metastatic MCC. While 81% (10/12) responded, this response was only durable for an average of 4 months.^8^ In a study population who received cisplatin and doxorubicin alone for SCC and BCC, the ORR was 68% (19/28) with a duration of 4 to 82 months. Only two of these patients remained in remission at the time of last follow-up.^9^ In general, immunotherapy is more commonly being used as monotherapy for unresectable NMSC; however, therapeutic risk and burden are higher, and not all patients will respond. A Phase II trial of 26 MCC patients receiving pembrolizumab for metastatic/locoregionally advanced disease showed a promising ORR of 56% (CR = 4, PR = 10), but 15% of patients had a Grade 3 or 4 adverse event, indicating serious or life threatening side effects of therapy.^10^ The Keynote-629 trial, a large, nonrandomized Phase II trial of pembrolizumab in locally advanced/metastatic SCC, showed an ORR of 50% for the locally advanced cohort (*n* = 54, CR = 9, PR = 18), and 35.2% in the recurrent/metastatic cohort (*n* = 105, CR = 11, PR = 26).^11^ Additionally, 11.9% of patients had a Grade 3-5 AE, including two deaths related to therapy. In a study by Gross et al., 51% of patients with Stage II-IV SCC achieved CR with two doses of cemiplimab. However, Grade 3-5 AEs occurred in 18% of patients, with one death attributable to treatment.^12^ While immune monotherapy is efficacious, intralesional therapy is an attractive alternative due to its relatively low risk and low treatment burden.^13^ At the same time, many of the aforementioned studies included patients with both in-transit and metastatic disease, whose therapeutic options and associated toxicity profiles may differ from our cohort with in-transit disease only. It is also important to note that alternative therapies exist for refractory in transit NMSC, such as isolated limb infusion and hyperthermic isolated limb perfusion, and demonstrate high efficacy, including PR of 50% in SCC and PR or CR of 60–92% in MCC patients.^14–17^ In contemporary therapy, this may be an attractive option for patients who are refractory to systemic immunotherapy and have particularly high burden of symptomatic disease, such as bulky, friable lesions that are difficult to inject or a high number of lesions that exceed what can realistically be injected.

T-VEC may also fill an important niche in the treatment of advanced NMSC in patients who have received organ transplants. While immune checkpoint inhibitors are playing an increasingly important role in the treatment of many NMSC, there are concerns that they may disturb the balance of immunologic tolerance in patients that have received organ transplants, putting them at risk of rejection.^19^ With cutaneous SCC in particular being a common cancer in the setting of chronic immunosuppression,^20^ the ability to create a more tumor-specific immune response using intralesional therapies may help patients to avoid this potentially serious adverse effect of immune checkpoint inhibitor therapy. Of note, experience using T-VEC in immunosuppressed patients is limited. Given case reports of disseminated HSV following administration of T-VEC in immune competent patients, we appreciate that this risk is undefined and may be higher in patients receiving chronic immune suppression.^23,24^

The efficacy of T-VEC in treating recurrent/in-transit melanoma was established in the OPTiM trial, which compared intralesional T-VEC to GM-CSF. In a final analysis of the findings of OPTiM, it was determined that the ORR of T-VEC in this cohort of patients with unresectable Stage IIIB-IVM1c melanoma was 31.5%.^18^ Of the patients who achieved CR (16.9%), 88.5% had a durable response at 5-year follow-up.^18^

The existing literature surrounding the use of T-VEC in NMSC has found similarly promising results. A meta-analysis of case reports, case series, and Phase I/II clinical trials in which patients received T-VEC for MCC (*N* = 8) found that six (75%) patients achieved partial response and two (25%) achieved CR. For patients who received T-VEC for SCC (*N* = 58), ten (16.1%) achieved partial response and four (6.4%) achieved CR.^6^ In the papers analyzed, only one MCC patient received concurrent T-VEC and IO, and only achieved CR after several cycles of IO without T-VEC. In the SCC cohort, no patients received concurrent immune therapy, and only three (4.8%) and six (9.6%) had received prior immune therapy and targeted biologics, respectively.^6^ Therefore, our results are unique in investigating the interaction between T-VEC and pembrolizumab in NMSC. We also anticipate results from an ongoing trial of T-VEC administered in combination with nivolumab, which will provide a more rigorous assessment for the utility and safety of this treatment strategy for NMSC.^21^

Although T-VEC was used in select cases of SCC, it was always used in conjunction with systemic immunotherapy, so it is not feasible to attribute any observed effects to T-VEC alone. Only one of three participants with SCC had a response while the remainder had progressive disease. Despite anticipating higher rates of response in patients receiving concurrent T-VEC and systemic immunotherapy, this was not observed. Compared with the results of the Keynote-629 trial, patients in this study who received combination T-VEC and pembrolizumab had an inferior ORR. This may be due to selection bias; patients with higher burden of disease were more likely to receive combination treatment while also being more likely to have an inferior outcome.

### Limitations

While NMSC do predominantly affect males, recent epidemiologic data suggests that approximately 40% of NMSC cases are diagnosed in women compared with 15.4% in our study.^22^ We also had a paucity of Black and Hispanic/Latino individuals represented. This lack of sex and racial diversity reduces the external validity of our findings. In addition, the cohort was 100% insured; 92.7% of patients were insured by Medicare. While T-VEC is typically covered by Medicare, coverage by other insurers is inconsistent and treatment may be prohibitively expensive for individuals who lack insurance altogether. Therefore, it is possible that insurance status at the time of consideration of T-VEC therapy impacted who was able to receive treatment and ultimately be a part of this study. Furthermore, the low utilization of T-VEC for NMSC due to lack of FDA approval or insurance authorization led to a study cohort that is small and underpowered to compare response rates for specific subgroups, such as those treated with or without concurrent immunotherapy.

Finally, the study is limited in its retrospective design as the data abstracted were not originally collected for research purposes, meaning that there is potential for variability in reporting among documenting physicians. Because patients were not enrolled under a study protocol, there is an inability to have standardized inclusion and exclusion criteria, efficacy measures (such as definition of response rate), or injection protocols. This lack of standardization has potential to influence outcomes based on variability in treatment received. There is also potential for selection bias in our study due to its nature as a retrospective cohort study. Because patients were not randomized into treatment groups, it is possible that physicians who used T-VEC, while exercising clinical judgement at the time of treatment, elected to give T-VEC to patients with specific tumor characteristics that may have been more or less favorable to achieving a response.

## Conclusions

Our data suggest that T-VEC may be a viable treatment for some patients experiencing recurrent or in-transit MCC or SCC that is refractory to standard-of-care therapies. We observed a high response rate and response durability among those who achieved a clinical complete response. However, due to the high rate of concurrent usage of immune therapy, it is difficult to attribute objective response to T-VEC alone. Additionally, our small sample size and paucity of female, Hispanic/Latino, and Black patients limits our ability to compare different groups and to apply our results to other contexts. Overall, our study highlights the need for a more rigorous exploration of the role T-VEC may play as a treatment option for advanced NMSC, with particular attention to its use with or without concurrent immune therapy.

## Supplementary Information

Below is the link to the electronic supplementary material.

Supplementary file1 (DOCX 19 KB)

## Data Availability

Cohort meta-data will be made available upon request. Individual patient data will not be made available to protect confidentiality.
