# Supplementary material for: Evaluation of Talimogene Laherparepvec for the Treatment of Advanced Nonmelanoma Skin Cancers
Source: Ann Surg Oncol. 2026 Feb 22;33(6):4985–90. doi: 10.1245/s10434-026-19182-3 (PMC13179203; doi:10.1245/s10434-026-19182-3)
Supplement: Supplementary file 1 — Supplementary file1 (DOCX 19 KB) [file 10434_2026_19182_MOESM1_ESM.docx]

**Supplemental Table.** Treatment modalities received by patient before and after T-VEC administration

| **ID** | **Cancer** | **Time from primary tumor diagnosis to TVEC (days)** | **Surgery prior to TVEC** | **Radiation prior to TVEC** | **Systemic therapy prior to TVEC** | **Con-**  **current IO** | **TVEC Cycles** | **Best Response** | **Response duration (days)** | **Vital status at follow-up** |
| --- | --- | --- | --- | --- | --- | --- | --- | --- | --- | --- |
| 1 | MCC | 157 | Yes | No | No | Yes | 10 | CR | 355 (ongoing) | Alive |
| 2 | MCC | 214 | Yes | No | No | No | 5 | CR | 627 (ongoing) | Alive |
| 3 | MCC | 209 | Yes | No | No | No | 8 | CR | 892 | Non-cancer death |
| 4 | MCC | 335 | Yes | Yes | No | No | 4 | CR | 1514 | Non-cancer death |
| 5 | MCC | 309 | Yes | No | No | No | 1 | CR | 288 (recurred) | Non-cancer Death |
| 6 | MCC | 1201 | Yes | Yes | No | Yes | 4 | PD | n/a | Alive |
| 7 | MCC | 100 | Yes | No | No | Yes | 1 | PD | n/a | Cancer death |
| 8 | MCC | 48 | Yes | No | No | Yes | 4 | PD | n/a | Cancer death |
| 9 | MCC | 238 | Yes | No | No | No | 3 | PD | n/a | Alive |
| 10 | MCC | 99 | Yes | No | No | No | 15 | PR | 1397 | Non-cancer death |
| 11 | SCC | 538 | Yes | Yes | Yes (IO) | Yes | 1 | CR | 106 (recurred) | Non-cancer death |
| 12 | SCC | 141 | No | No | Yes (Chemo) | Yes | 4 | PD | n/a | Alive |
| 13 | SCC | 207 | Yes | No | Yes (IO) | Yes | 1 | PD | n/a | Cancer death |

MCC=Merkel cell carcinoma; SCC=Squamous cell carcinoma; IO=immunotherapy; CR=complete response; PD=progressive disease; PR=partial response

**Supplemental Table 2.** Burden of disease including maximum lesion size, number of lesions treated, and volume of T-VEC administered at initial visit.

| ID | Number of lesions treated | Maximum lesion diameter (centimeters) | Volume of T-VEC injected (milliliters) |
| --- | --- | --- | --- |
| 1 | 2 | 3.5 | 4 |
| 2 | 3 | 1.2 | 2 |
| 3 | 2 | 5.1 | 3 |
| 4 | 7 | 1.2 | 4 |
| 5 | 4 | 1 | 2 |
| 6 | 1 | 2 | 2 |
| 7 | 15 | 2.5 | 3 |
| 8 | 2 | 3.5 | 2 |
| 9 | 1 | 1.4 | 1 |
| 10 | 4 | 3 | 2 |
| 11 | 1 | 5 | 2 |
| 12 | 5 | 2.5 | 4 |
| 13 | 8 | 3 | 3 |
